# Profil épidémiologique et prise en charge des exacerbations d'asthme chez l'enfant à l'hôpital d'enfants de Rabat au Maroc

**DOI:** 10.11604/pamj.2015.20.73.4031

**Published:** 2015-01-28

**Authors:** Mohamed El Mahdi Boubkraoui, Fadoua Benbrahim, Abdellah Assermouh, Naima El Hafidi, Soumia Benchekroun, Chafiq Mahraoui

**Affiliations:** 1 Unité de Pneumoallergologie Pédiatrique, Hôpital d'Enfants de Rabat, CHU Ibn Sina, Faculté de Médecine et de Pharmacie, Université Mohamed V Souissi, Rabat, Maroc

**Keywords:** Asthme, exacerbation, asthme aigu grave, enfant, épidémiologie, Asthma, exacerbation, acute severe asthma, child, epidemiology

## Abstract

**Introduction:**

L'exacerbation d'asthme est un phénomène paroxystique qui peut mettre en jeu le pronostic vital. Le but de l’étude est d’évaluer le profil épidémiologique et les modalités de prise en charge de l'exacerbation d'asthme chez les enfants âgés de 2 à 15 ans dans l'unité de pneumoallergologie pédiatrique de l'hôpital d'enfants de Rabat au Maroc.

**Méthodes:**

Il s'agit d'une étude rétrospective qui a concerné 1461 enfants hospitalisés pour exacerbation d'asthme modérée à sévère durant une période d'un an allant de décembre 2011 à novembre 2012, les exacerbations légères étant traitées en ambulatoire.

**Résultats:**

Les hospitalisations pour exacerbation d'asthme chez les enfants ont représenté 34% de l'ensemble des hospitalisations avec trois pics en mai, septembre et décembre. L’âge moyen de survenue était de 3 ans et demi avec une prédominance masculine nette. L'exacerbation d'asthme était inaugurale dans 22% des cas. Les infections respiratoires virales dominaient les facteurs déclenchants des exacerbations d'asthme. Le séjour hospitalier était en moyenne de 3 jours. Un transfert en réanimation a été nécessaire dans 2% des cas. L’évolution sous traitement a toujours été favorable et la mortalité a été nulle.

**Conclusion:**

La prévalence des hospitalisations pour exacerbation d'asthme suit un profil saisonnier lié aux effets environnementaux. La plupart de ces hospitalisations pourraient cependant être évitées grâce à un meilleur contrôle de l'asthme et à l'amélioration de l’éducation thérapeutique de l'enfant asthmatique et de son entourage.

## Introduction

L'asthme est une maladie inflammatoire chronique des voies aériennes entrainant une obstruction réversible et une hyperréactivité des voies aériennes à divers stimuli avec des symptômes récidivants. C'est la maladie chronique la plus fréquente en pédiatrie. Sa prévalence chez l'enfant au Maroc est de l'ordre de 10 à 15% avec une augmentation croissante [1]. Les exacerbations d'asthme sont des phénomènes paroxystiques avec des symptômes aigus se prolongeant au-delà de 24 h et qui peuvent mettre en jeu le pronostic vital. Elles constituent au moins 5% des urgences de l'enfant [2]. Le but de cette étude est d’évaluer le profil épidémiologique ainsi que les modalités de prise en charge de l'exacerbation d'asthme chez les enfants âgés de 2 à 15 ans dans l'unité de pneumoallergologie pédiatrique de l'hôpital d'enfant de Rabat au Maroc.

## Méthodes

Il s'agit d'une étude rétrospective, descriptive et transversale qui a concerné les dossiers d'enfants âgés de 2 à 15 ans qui ont été hospitalisés pour exacerbation d'asthme modérée à sévère dans l'unité de pneumoallergologie pédiatrique de l'hôpital d'enfant de Rabat au Maroc durant une période d'un an, allant de décembre 2011 à novembre 2012. Les exacerbations légères d'asthme ont été prises en charge en ambulatoire. L'analyse a porté au total sur 1461 patients et les paramètres suivants ont été analysés pour chaque patient: date d'hospitalisation, âge et sexe, antécédents, histoire de la gêne respiratoire, données de l'examen clinique initial et traitement entrepris.

L’évaluation de la sévérité de l'exacerbation d'asthme s'est basée sur les recommandations du GRAPP [2]. L'existence d'une atopie personnelle chez les patients a été retenue devant l'existence d'une rhinite allergique, d'une dermatite atopique ou d'une conjonctivite chez l'enfant. L'existence d'une atopie familiale a été retenue devant l'existence d'un asthme, d'une rhinite allergique ou d'un eczéma chez les parents ou la fratrie. L'existence d'une exposition allergénique a été retenue devant la présence de tapis, moquettes, animaux, plantes, blattes ou moisissures dans le lieu de vie du patient. L'existence d'un tabagisme passif a été retenue en cas d'exposition au tabagisme parental. L'existence d'une obésité a été retenue lorsque l'indice de masse corporelle était supérieur au 90^e^ percentile sur les courbes de corpulence. L'existence d'un reflux gastro-œsophagien a été retenue devant les données de la pH-métrie de 24 h. Une infection virale a été suspectée comme étant le facteur déclenchant lorsqu'une symptomatologie d'allure virale avait précédé la survenue de l'exacerbation d'asthme. Le contrôle de l'asthme a été évalué selon les critères proposés par GINA 2006 et NAEPP 2007 [3, 4].

Les patients ont été traités selon le protocole du service: prednisolone à la dose de 2 mg/kg/jour sans dépasser 60 mg/jour pendant 5 jours, nébulisations avec une source d'oxygène à 6 l/min de salbutamol à la dose de 0,15 mg/kg avec un minimum de 1,5 mg et sans dépasser 5 mg (jusqu’à 6 séances de nébulisations espacées de 20 min puis toutes les 4 h) et oxygénothérapie en cas de saturation en oxygène inférieure à 93%. Une éducation thérapeutique a été proposée aux enfants et à leurs parents durant le séjour hospitalier, généralement au deuxième jour de l'hospitalisation lors d'une session d’école de l'asthme. À la sortie, les patients ont reçu un traitement de consolidation: 2 bouffées de salbutamol inhalé à répéter 4 fois par jour pendant 5 jours et corticothérapie orale pour compléter 5 jours de traitement.

## Résultats

**Figure 1:**
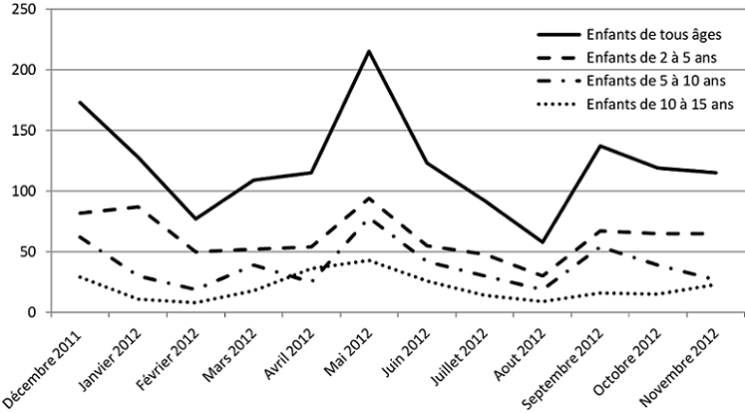
Nombre d'hospitalisations pour exacerbation d'asthme au cours de l'année par classe d’âge

Durant la période de l’étude, les exacerbations d'asthme chez les enfants âgés de 2 à 15 ans ont représenté 34% des motifs d'hospitalisation dans l'unité de pneumoallergologie pédiatrique et 8% des motifs d'hospitalisation dans l'ensemble de l'hôpital d'enfants de Rabat. Des pics d'hospitalisations ont été constatés en mai, septembre et décembre. Le minimum d'hospitalisations a été observé au mois d'aout (Figure 1). Durant la même période, 3% des enfants on été réadmis au moins une fois pour le même motif et 37% des réadmissions sont survenue dans les 7 jours suivant la première hospitalisation.

Les caractéristiques de la population étudiée sont consignées dans le Tableau 1. L’âge moyen des patients était de 3,5 ans et le sexe-ratio était de 1,5. Un antécédent d'oxygénothérapie à la naissance pendant une durée variant de 1 à 26 jours avec une moyenne de 4 jours a été retrouvé chez 4% des enfants. Une atopie personnelle a été retrouvée dans 30% des cas et une notion d'atopie familiale a été retrouvée chez 43% des patients. L'exposition allergénique concernait 37% des enfants. Une exposition au tabagisme passif existait dans 18% des cas et le tabagisme actif était nul. Les patients étaient originaires de la ville de Rabat dans 49% des cas. Ils étaient originaires des deux villes bordant Rabat (Salé et Témara), dans respectivement 26% et 17%. Les autres patients étaient originaires de régions proches de Rabat dans 5% des cas et ils provenaient de régions plus éloignées dans 3% des cas.

**Table 1:** Caractéristiques de la population étudiée

Caractéristiques	Effectif	Pourcentage
Sexe masculin	590	40%
Sexe féminin	871	60%
Âge entre 2 et 5 ans	749	51%
Âge entre 5 et 10 ans	464	32%
Âge entre 10 et 15 ans	248	17%
Antécédent de prématurité	63	4%
Oxygénothérapie à la naissance	59	4%
Rhinite allergique	267	18%
Conjonctivite	177	12%
Dermatite atopique	58	4%
Atopie familiale	623	43%
Exposition allergénique	536	37%
Tabagisme passif	270	18%
Obésité	33	2%
Reflux gastro-œsophagien	24	2%

L'exacerbation d'asthme était inaugurale chez 22% des enfants dont 79% étaient âgés de 2 à 5 ans, 19% étaient âgés de 5 à 10 ans et 2% étaient âgés de 10 à 15 ans. Le délai de consultation aux urgences de l'hôpital d'enfants de Rabat après le début de l'exacerbation d'asthme variait de quelques heures à 7 jours avec une moyenne de 1,8 jour. Les patients s’étaient présentés aux urgences d'une autre formation hospitalière dans les heures ou jours précédents leurs hospitalisation dans 16% des cas. Avant leur admission, 12% des patients avaient reçu un traitement bronchodilatateur seul et 28% des patients avaient reçu un traitement bronchodilatateur et une corticothérapie orale. L'exacerbation d'asthme était modérée à sévère dans 98% des cas. Un asthme aigu grave a nécessité un transfert en réanimation dans 2% des cas.

Une infection virale a été suspectée dans le déclenchement de l'exacerbation d'asthme chez 75% des enfants. Un reflux gastro-oesophagien était associé à l'asthme chez 2% des enfants. Une obésité a été retrouvée chez 2% des enfants. Un traitement de fond à base de corticothérapie inhalée ou corticothérapie inhalée associé à un bêta-2-mimétique inhalé à longue durée d'action était institué chez 27% des enfants et durant les 3 mois précédant l'exacerbation, 12% des enfants avaient arrêté leur traitement de fond.

Tous les enfants ont été traités selon le protocole du service avec une corticothérapie per os et des nébulisations de salbutamol. Le recours à l'oxygénothérapie a été nécessaire chez 6% des patients. L’évolution a été favorable chez tous les patients et aucun cas de décès n'a été enregistré dans notre série. Le séjour hospitalier variait de quelques heures à 15 jours, il était en moyenne de 3 jours et ne variait pas selon qu'il s'agissait ou non d'une exacerbation d'asthme inaugurale et selon que les patients recevaient ou non un traitement de fond de l'asthme. Tous les patients ont reçu un traitement de consolidation à la sortie. Un traitement de fond de l'asthme a été instauré ou renforcé chez 35% des enfants qui présentaient des critères de non contrôle de l'asthme. Un plan d'action à adopter en cas d'exacerbation d'asthme dument expliqué a été remis par écrit à tous les enfants et à leurs parents. Des explications leurs ont été également prodiguées sur le mode d'emploi du matériel d'inhalation. Enfin, tous les patients ont été adressés en consultation spécialisée de pneumoallergologie pédiatrique pour prise en charge de leur asthme.

## Discussion

Les exacerbations d'asthme chez l'enfant ont représenté une proportion importante des motifs d'hospitalisation à l'hôpital d'enfants de Rabat durant la période de l’étude avec 3 pics constatés durant l'année. Le pic du mois de décembre coïncide avec la recrudescence des infections respiratoires virales, notamment à rhinovirus [5]. Le pic du mois de mai, plus prononcé chez les enfants âgés de 5 à 15 ans, coïncide avec le maximum de pollen saisonnier [6]. Le pic du mois de septembre coïncide avec la rentrée scolaire et la reprise de la vie en collectivité avec une recrudescence des infections respiratoires virales, auxquels se rajouterait parfois l'arrêt du traitement de fond pendant les vacances [7].

L’âge moyen des patients était de 3,5 ans et plus que la moitié d'entre eux était âgé de 2 à 5 ans. Cette proportion élevée d'hospitalisations pour cette tranche d’âge peut être expliquée par le seuil bas d'apparition de la détresse respiratoire qui est en rapport avec la petite taille des voies aériennes chez les jeunes enfants. Ces derniers présentent par conséquent des exacerbations d'asthme plus sévères, entrainant des hospitalisations plus fréquentes.

La prédominance masculine était évidente dans notre série. Plusieurs études épidémiologiques ont noté une proportion plus élevée des hospitalisations pour exacerbation d'asthme chez les garçons avant la puberté puis une inversion du sexe-ratio entre 15 et 18 ans [8–11]. L'argument des influences hormonales a été avancé pour expliquer ces différences. Certaines études suggèrent cependant qu'il existe une réduction de l’écart du taux d'hospitalisations pour exacerbation d'asthme entre les deux sexes pendant l'enfance [12].

L'association asthme et rhinite allergique est fréquente [13]. Presque un cinquième des patients dans notre série présentaient une rhinite allergique. À ce propos, certaines études suggèrent que le traitement de la rhinite allergique avec des corticostéroïdes nasaux améliore le contrôle de l'asthme chez les enfants et pourrait par conséquent diminuer le risque d'exacerbation d'asthme [14].

Le rôle des infections respiratoires virales dans la survenue des exacerbations d'asthme chez l'enfant a été largement décrit. Et bien que l'association entre exacerbation d'asthme et infection virale n'a pas été prouvée dans cette étude, d'autres travaux ont prouvé que les infections virales étaient liées à dans une grande proportion aux exacerbations d'asthme [5, 9]. Une augmentation du risque d'exacerbation d'asthme par une interaction synergique entre infections respiratoires virales et exposition allergénique a également été mise en évidence [15].

L'inobservance du traitement de fond de l'asthme constitue un facteur de survenue d'exacerbation d'asthme [16]. Dans notre étude, 12% des enfants avaient arrêté leur traitement de fond dans les 3 mois précédents l'exacerbation d'asthme, indiquant que tous les patients n'avaient pas reçu une éducation thérapeutique avec un niveau de sécurité suffisant. Une éducation thérapeutique bien menée et continue doit impérativement être intégrée au traitement de l'asthme puisqu'elle a montré son efficacité dans la prévention des exacerbations d'asthme en diminuant le recours aux urgences et aux hospitalisations [17–19].

La prise en charge des exacerbations d'asthme en milieu hospitalier est bien codifiée. Les nébulisations de salbutamol, la corticothérapie et l'oxygénothérapie à la demande sont la base du traitement dans notre contexte. Le séjour hospitalier moyen chez la population étudiée était de 3 jours. L'exacerbation d'asthme cède en effet rapidement lorsqu'une thérapeutique efficace est appliquée rapidement, permettant un séjour hospitalier bref. L'administration précoce de la corticothérapie systémique dès l'admission permet notamment la réduction de la durée d'hospitalisation [20].

Dans tous les cas, le recours à l'hospitalisation pour exacerbation d'asthme doit être considéré comme un témoin de la gravité de la maladie et un indicateur d'une prise en charge non optimale, même si une partie des hospitalisations semble être inévitable. L'hospitalisation pour exacerbation d'asthme doit donc constituer une opportunité pour réévaluer l'adéquation de la prise en charge de l'enfant asthmatique. Une réduction des réadmissions pour exacerbation d'asthme peut être obtenue par l'instauration ou l'adaptation du traitement de fond, le contrôle de l'environnement, la remise d'un plan d'action écrit, l’éducation thérapeutique dispensée dans une école de l'asthme et le suivi en consultation spécialisée de pneumoallergologie pédiatrique [21]. Il faut en particulier lutter contre la corticophobie et résoudre les difficultés techniques liées à la voie d'administration inhalée. Sans cette réévaluation de la prise en charge, la survenue d'une exacerbation d'asthme constituera un fort facteur prédictif d'une future exacerbation avec un risque de réadmission dans l'année plus accru chez les très jeunes enfants et chez les enfants issus d'un milieu socioéconomique défavorisé [22–24].

## Conclusion

La prévalence des hospitalisations pour exacerbation d'asthme chez l'enfant suit un profil saisonnier lié aux effets environnementaux. La plupart de ces hospitalisations pourraient cependant être évitées grâce à un meilleur contrôle de l'asthme et à l'amélioration de l’éducation thérapeutique de l'enfant asthmatique et de son entourage. Ceci passe par la mise à leur disposition d'un plan d'action permettant une adaptation précoce du traitement dès la reconnaissance des signes d'exacerbation. Le but étant de diminuer la morbidité, l'absentéisme scolaire et le cout très élevé des hospitalisations pour exacerbation d'asthme chez l'enfant.
